# A Widening Scope

**Published:** 1914-07

**Authors:** 


A Widening Scope.—There is nothing more significant of the progress of the medical profession than the action of the last convention of the American Medical Association. That convention was not merely a meeting of physicians to discuss abstruse technical subjects, but was an active body of some four thousand physicians earnestly at work in the service of mankind. It is a hopeful sign when professional men in their gatherings give more time to studying how they can improve existing evils than they give to discussing things that may bring them in greater revenue. Dr. W. C. Vaughan, the newly elected President of the Association, says that a definite policy for the coming year has been outlined, and that the time and money of the Association will be given to educating the public along lines tending to better conditions and to prolong human life.

A few years ago and the physician who devoted his best attention to curing the ills of his patient regarded himself as in the discharge of his sole duty. In rare cases he might perhaps advise patients how to avoid future illness, but counsel of this kind was given exclusively to those who sought his medical advice. Now, physicians everywhere have become active in every effort to bring about conditions for minimizing sickness and prolonging life. They do this work unselfishly, too, for at first thought it would appear that physicians prosper most when people are oftenest in need of their services. However strange it may appear, the best physicians have never before been quite so prosperous, nor the people quite so healthy.

Another good sign of progressiveness is the fact that large sums of money are to be spent in securing articles for the newspapers—articles that will teach the people how to preserve their health. Not so very long ago the profession thought it wise to profoundly guard its professional information and withhold it from the public, and newspaper publicity was tabooed as a thing wholly unethical. A new code of ethics appears to be growing up in the profession—a code that recognizes the practice of medicine as an opportunity for rendering great public service, and for doing the most good to the greatest number of people. It is a high standard for some to comprehend, but one towards which the most eminent practitioners are rapidly coming.

